# A Large Language Model–Based System for Depression Symptom Identification: Iterative Qualitative Co-Design Studies

**DOI:** 10.2196/95422

**Published:** 2026-11-03

**Authors:** Abeer Alotaibi, Corina Sas, Hossein Rahmani

**Affiliations:** 1 School of Computing and Communication, Lancaster University, InfoLab21, South Dr, Bailrigg, Lancaster, England, LA1 4WA, United Kingdom, 44 7787408223

**Keywords:** mental health, large language models, AI, HCI, depression, symptom identification, co-design, human-computer interaction

## Abstract

**Background:**

AI technologies have considerable potential to support mental health practitioners in identifying depression symptoms. However, existing tools are often developed with limited practitioner involvement and evaluated primarily on technical performance, leaving questions of clinical relevance, ethical design, and practitioner trust insufficiently addressed.

**Objective:**

This study aimed to examine how iterative co-design with mental health practitioners can inform the development of an AI-based model for identifying depression symptoms, focusing on how practitioner input shaped its clinical relevance and ethical foundations across 2 design cycles.

**Method:**

We conducted a 2-stage qualitative co-design study. In stage 1, we developed DepressionSymp 1.0, a fine-tuned GPT-3.5 model trained on the CounselChat dataset, identifying 5 symptom categories based on the *Diagnostic and Statistical Manual of Mental Disorders, Fifth Edition* (*DSM-5*) criteria and the Linguistic Inquiry and Word Count (LIWC-22) psycholinguistic framework, reviewed and discussed by 6 mental health practitioners during online co-design workshops. In stage 2, this feedback informed DepressionSymp 2.0, expanded to 10 symptom categories and reviewed by 8 further practitioners in a second round of workshops.

**Results:**

Practitioners valued the structured symptom outputs for reducing cognitive workload and supporting intervention planning. In stage 1, indirect indicators such as social withdrawal, financial difficulties, and major life changes were often classified as lifestyle factors rather than symptoms; this informed the expansion to 10 categories in stage 2, described as clearer and more useful for preassessment. Risk categorization was identified as the most ethically sensitive aspect across both stages, alongside broader concerns including the lack of nonverbal information in text-based assessments, cultural and linguistic differences in symptom expression, and the potential for automation bias without sufficient human oversight.

**Conclusions:**

Iterative co-design with mental health practitioners is valuable for developing AI-based tools that are clinically relevant and ethically grounded, demonstrating how qualitative practitioner feedback can inform iterative model refinement and ethical design. Findings inform design implications around large language model (LLM) features, practitioner-in-the-loop oversight, and ethical considerations covering clinical standards, informed consent, and data governance. Future work should prioritize larger-scale independent validation, formal safety evaluation, and culturally diverse dataset development.

## Introduction

AI-human-computer interaction (AI-HCI) research has seen growing interest across mental health applications, using machine learning [1], speech analysis [2], natural language processing (NLP) [3], or large language models (LLMs) [4] to support tracking patient status [5], identifying symptoms [6], classifying disorders [7], clinical decision-making [8-10], and personalized interventions [11]. Systematic reviews confirm AI’s value for detecting symptoms and risk factors, diagnosis, and personalized intervention across text, speech, image, and biosensor data [12], while also highlighting the field’s prevalent focus on automated diagnosis and its ethical challenges [13]. AI-based recommendations for personalized interventions, such as mobile apps [14] and stress-management recommender systems [15], tend to argue for limited practitioner presence [16].

On symptom identification specifically, recent work has explored AI’s potential to extract and analyze information from user-generated content, clinical notes, or structured assessments [17-20]. NLP-based models have proven effective for identifying symptoms of depression and anxiety through text [21,22]; sentiment analysis tools and conversational agents, for instance, detect emotional and psychological markers from word choice and response patterns in social media posts or online forums [23,24]. Such work is often trained on benchmark datasets such as CLPsych, based on Twitter data [25], and several Reddit- or Twitter-based corpora supporting binary or multilabel classification of depression-related states [26-30]. Clinical datasets, such as Distress Analysis Interview Corpus – Wizard-of-Oz (DAIC-WOZ) and its extension Extended Distress Analysis Interview Corpus (E-DAIC), are used less often due to privacy restrictions and limited access, relying instead on structured interviews rather than freely expressed language [31-33].

Building on this work, LLMs have recently emerged as a promising direction for extracting and organizing symptom-related information from clinical text, with recent work summarizing clinical dialogues showing strong text-similarity scores against reference summaries, confirmed by both clinicians and nonclinicians [34]. Other work has applied LLMs to screening and diagnostic reporting, including classifying depressive states from synthetic interviews [35], generating *Diagnostic and Statistical Manual of Mental Disorders, Fifth Edition* (*DSM-5*)-based diagnostic reports from posttraumatic stress disorder (PTSD) interviews [36], predicting severity from patient dialogue [37], producing multiclass outputs for stress, depression, and suicidality [38], and combining doctor-style replies with symptom summaries [39,40].

Despite these advances, 3 important gaps remain. First, depression’s complexity and symptom overlap challenge accurate AI classification [41], with existing models often lacking a clear clinical rationale for their categorization approaches [8,42-44] and depending on patients expressing themselves honestly, as concealment or exaggeration may harm assessment validity [45]. Second, the ethical implications of these models remain underexplored despite growing human-computer interaction (HCI) interest in health-technology ethics [13,46], particularly given the impact on vulnerable individuals; ethical LLMs are designed and evaluated against principles such as accuracy, reliability, interpretability, and trust [47-49], yet limited work explores practitioners’ perspectives on these in mental health contexts. Third, most models have been developed with limited practitioner input, despite this being crucial for model validity, accuracy, and clinical integration [44,50-54].

To address this, we present the iterative co-design of DepressionSymp, an LLM-based model for identifying depression-related symptoms from text narratives, conducted in 2 stages: DepressionSymp 1.0 was developed and qualitatively evaluated through workshops with 6 mental health practitioners, with insights informing DepressionSymp 2.0, explored with 8 further practitioners. Across both stages, practitioner feedback was structured around 4 ethical principles: accuracy, comparing practitioners’ symptom identification with model outputs; reliability, examining output consistency; interpretability, exploring whether practitioners could understand and act on outputs; and trust, capturing confidence in the system’s suitability for clinical use (Figure 1).

Our work makes the following contributions: (1) rich qualitative accounts of mental health practitioners’ perspectives that informed the iterative design of our LLM-based DepressionSymp models; (2) their ethical concerns related to accuracy, interpretability, reliability, and trust; and (3) design implications for future LLM-based mental health systems across 3 areas: LLM features supporting contextual understanding, cultural interpretation, and risk evaluation; practitioner-in-the-loop oversight through iterative co-design; and ethical considerations around clinical standards, informed consent, and data governance. This is significant, as models can be technically functional yet clinically rejected if practitioners cannot interpret or trust their outputs. Our work focuses on three research questions (RQs):

1.  RQ1: How accurate and ethical are the DepressionSymp models for identification of depression symptoms?
2.  RQ2: How can we co-design with mental health practitioners, and what is the value of their input for the development of LLM-based models for identifying depression symptoms?
3.  RQ3: What are the ethical concerns associated with LLM-based models for identifying depression symptoms in clinical practice, and how could these be addressed?

**Figure 1.**
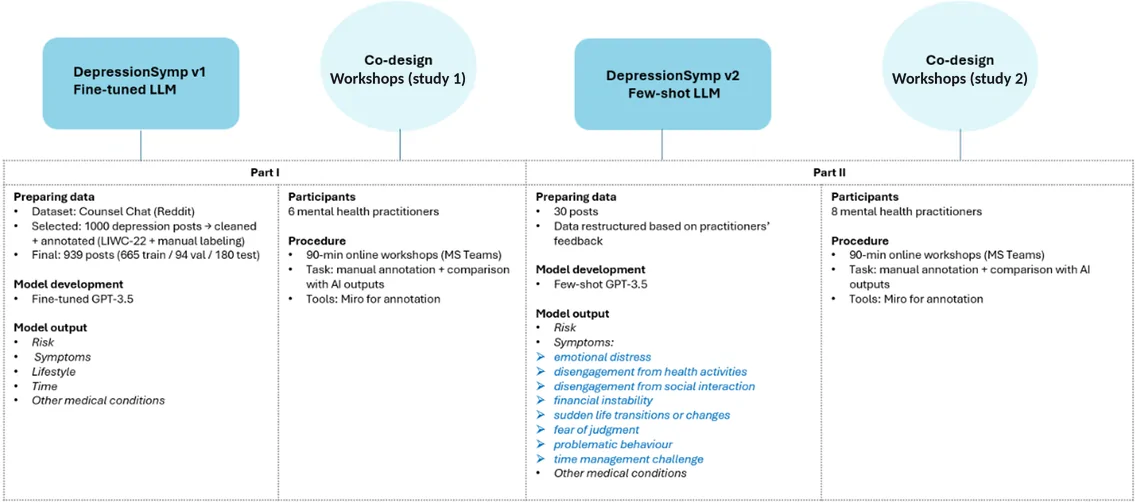
DepressionSymp model starting with 1.0 with fine-tuned large language model (A) and its exploration through the co-design workshops in study 1 (B), followed by the development of DepressionSymp 2.0 with few-shot prompting (C), and its exploration through co-design workshops in study 2. LIWC: Linguistic Inquiry and Word Count; LLM: large language model.

## Study 1

### Methods

#### Overview

This study uses a qualitative approach using iterative co-design workshops. The model was first designed and tested using recall-oriented understudy for gisting evaluation (ROUGE) scores against reference summaries, then explored by practitioners through co-design workshops to evaluate and refine both models.

#### Data Preparation

To train our model, we used the CounselChat dataset [55], a publicly available Reddit dataset organized into 5 mental health topics: addiction, anxiety, depression, eating disorders, and suicide. It was selected as a depression-specific, publicly accessible source of freely written posts capturing both emotional experiences and contextual factors, whereas access to naturalistic clinical conversations is often restricted by privacy requirements [56]. From the depression subset (114,081 posts), we randomly selected 1000 posts for manual annotation, sampling approximately one-third from each of 3 length groups (short: 50‐150 words; medium: 151‐300 words; and long: 301‐450 words) to reduce length-related bias [44,57,58].

Given the acknowledged bottleneck in engaging health experts for large corpus annotation [59], the lead researcher conducted the annotation of 1000 posts for training the first model. Practitioners also independently annotated 30 posts from the testing set and compared their assessments with the model outputs, enabling qualitative comparison between practitioner and model outputs and informing iterative refinement of the system during the co-design process. The lead researcher’s annotation was informed by the *DSM-5* depression criteria [60] and the Linguistic Inquiry and Word Count (LIWC-22), a validated psycholinguistic tool categorizing words into emotional, cognitive, and social domains [61].

The *DSM-5* is the main mental health guide providing standard diagnostic criteria [60], also used by LLMs [62]. Guided by its core criteria for depression, we mapped relevant constructs to LIWC categories as follows: (1) symptoms, that is, negative emotions such as sadness, anger, or fatigue, and positive emotions such as joy, pleasure, or interest; (2) risk, that is, suicidal thoughts; (3) time, that is, months, years, and days; (4) lifestyle, that is, work, home, leisure, wellness, food, and social relationships including family and friends, and other conditions requiring clinical attention; and (5) physical health and ill-health, that is, serious medical illness or disability [63]. Table 1 illustrates how each DepressionSymp 1.0 category is grounded in both *DSM-5* diagnostic criteria and LIWC-22 psycholinguistic constructs.

**Table 1.** DepressionSymp 1.0 categories mapped to *DSM-5^a^* diagnostic criteria and LIWC-22^b^ psycholinguistic constructs: symptoms to core emotional criteria (depressed mood and loss of interest) and negative and positive emotion categories; risk to suicidal ideation criteria and risk and death categories; time to duration and persistence requirements and time orientation categories; lifestyle to conditions for further clinical attention (occupational, social, environmental factors) and work, home, school, and social categories; and medical conditions to comorbidity criteria and physical health and ill-health categories.

DepressionSymp 1.0	*DSM-5*	LIWC-22
Symptoms	Negative emotions	Negative and positive emotion
Risk	Suicidal thoughts	Risk and death
Time	Period	Time orientation
Lifestyle	Focus of clinical attention	Work, home, school, and social
Medical conditions	Comorbidity	Physical health and ill-health

a *DSM-5*: *Diagnostic and Statistical Manual of Mental Disorders, Fifth Edition*.

b LIWC-22: Linguistic Inquiry and Word Count.

The annotation process involved careful review of the surrounding text. For each LIWC-identified word, we (1) verified that the assigned LIWC category reflected its intended meaning and (2) included nearby words when they were needed to interpret the context, such as temporal expressions (eg, all the time, never) and intensity terms (eg, very, highly, and super). Following LIWC-22–based extraction, the lead researcher manually reviewed each word within its sentence or paragraph to confirm the assigned category and ensure annotations captured meaningful sentence-level context rather than isolated keywords. During this process, 61 irrelevant or off-topic posts (eg, advertisements) were removed, leaving 939 posts for annotation, evenly distributed across the 3 post length groups. These were split into 665 training, 94 validation, and 180 test examples.

#### Model Development: DepressionSymp 1.0

We used supervised fine-tuning to adapt the GPT-3.5 model [64] in order to extract structured mental health information from user texts. GPT-3.5 was selected for DepressionSymp 1.0 because, at the time of development, GPT-4 and other newer models applied stricter safety restrictions limiting fine-tuning on sensitive content such as self-harm and suicidal ideation, which is essential to this task, whereas GPT-3.5’s fine-tuning API was more permissive for this type of clinical text. Alternative models such as DeepSeek were not selected due to documented hallucination concerns raised in independent evaluations [65,66]. In this approach, the model is trained on annotated examples that represent correct outputs for given inputs, using the manually labeled posts described in the Data Preparation section above. Each example was labeled with categories relevant to mental health, including symptoms, risk, time, lifestyle indicators, and other medical conditions (Figure 2). The model was fine-tuned using OpenAI’s API, with hyperparameters automatically configured according to OpenAI’s recommended defaults for small datasets (3 epochs, batch size of 1, and learning rate multiplier of 2) to optimize performance given the dataset size. Figure 2 shows an example of DepressionSymp 1.0, illustrating a user’s input post (left) and the model’s structured output (right).

**Figure 2.**
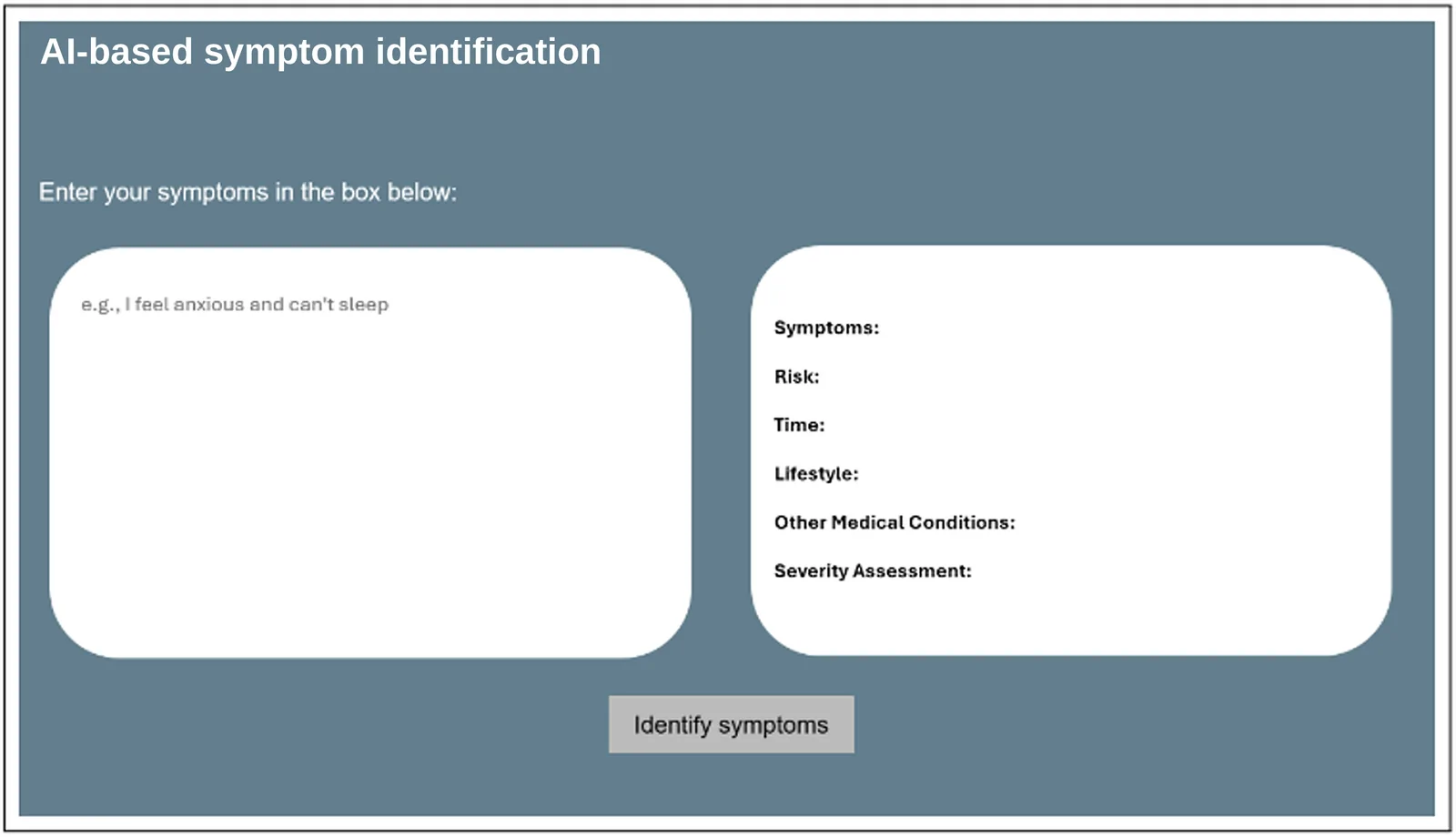
DepressionSymp 1.0 model showing (left) a user’s input post describing mental health experiences, and (right) the model’s structured output.

#### Model Testing

The model was evaluated using the ROUGE metric [67], which measures the quality of generated summaries by comparing them with reference summaries. ROUGE-1 evaluates the overlap of unigrams (individual words) between the generated summary and the reference [68]. ROUGE-2 measures the overlap of bigrams (consecutive word pairs) and ROUGE-L measures the longest common subsequence (LCS) between the generated and reference summaries [69]. ROUGE scores were chosen to evaluate text similarity and the model’s ability to extract exact linguistic expressions from patient text, preserving words and nuances. This approach captures subtle differences in wording and intensity, accurately reflecting patients’ expressions [70,71]. Table 2 presents the ROUGE scores for the zero-shot and fine-tuned GPT-3.5 model.

**Table 2.** ROUGE^a^ scores comparing zero-shot GPT-3.5 performance against the fine-tuned DepressionSymp 1.0 model. It shows precision, recall, *F*_1_-score, and percentage improvement across ROUGE-1, ROUGE-2, and ROUGE-L metrics.

ROUGE	Zero-shot	Precision	Recall	*F*_1_-Score	Improvement (%)
ROUGE-1	0.6032	0.9077	0.9323	0.9138	31
ROUGE-2	0.4787	0.8750	0.8979	0.8807	40
ROUGE-L	0.4705	0.8674	0.8901	0.8730	40

a ROUGE: recall-oriented understudy for gisting evaluation.

These results indicate the value of fine-tuning in this context: although GPT-3.5 showed some capability in a zero-shot setting, its performance remained limited (ROUGE-1: 0.60), suggesting that additional training on domain-specific annotated data was needed to consistently identify and structure depression-related symptoms aligned with *DSM-5* criteria.

#### Participants

Participants were recruited through counseling directories and contacted by email with study details; those who were accredited only and interested received a consent form and information sheet. The evaluation involved 6 participants (2 male and 4 female), aged 25‐35, with 2‐10 years of professional experience across clinical and nonclinical mental health settings, drawing on modalities including cognitive behavioral therapy (CBT), psychodynamic, person-centered, and trauma-informed approaches. While our purposive sample size was limited to 6 practitioners for study 1, we argue for its adequacy [72]. This is grounded in the high expertise we deliberately recruited, and the hands-on co-design activities which together have ensured “information power” or data richness [73]. In particular, given that our participants were accredited practitioners assessing a specific model’s outputs against their own clinical judgment, a task involving narrow and expertise-dense evaluation, a sample of 6 was judged adequate to generate actionable design feedback for model revision. Full demographic and professional details for each participant are provided in Multimedia Appendix 1. Workshops for study 1 were conducted in February 2025.

#### Workshop Procedure

Both studies were evaluated through individual 90-minute online workshops conducted via Microsoft Teams, audio-recorded and transcribed, with all participant data anonymized before analysis. Each participant was assigned 5 posts of varying lengths and asked to manually identify symptoms and other relevant mental health factors using the Miro platform (Figure 3), with their assessments then compared to the AI model outputs. Semistructured interviews explored the 4 ethical principles, ethical challenges related to the model outputs, and recommendations for model refinement through the co-design process. See Multimedia Appendix 2 for further detail on the workshop procedure and interview questions.

**Figure 3.**
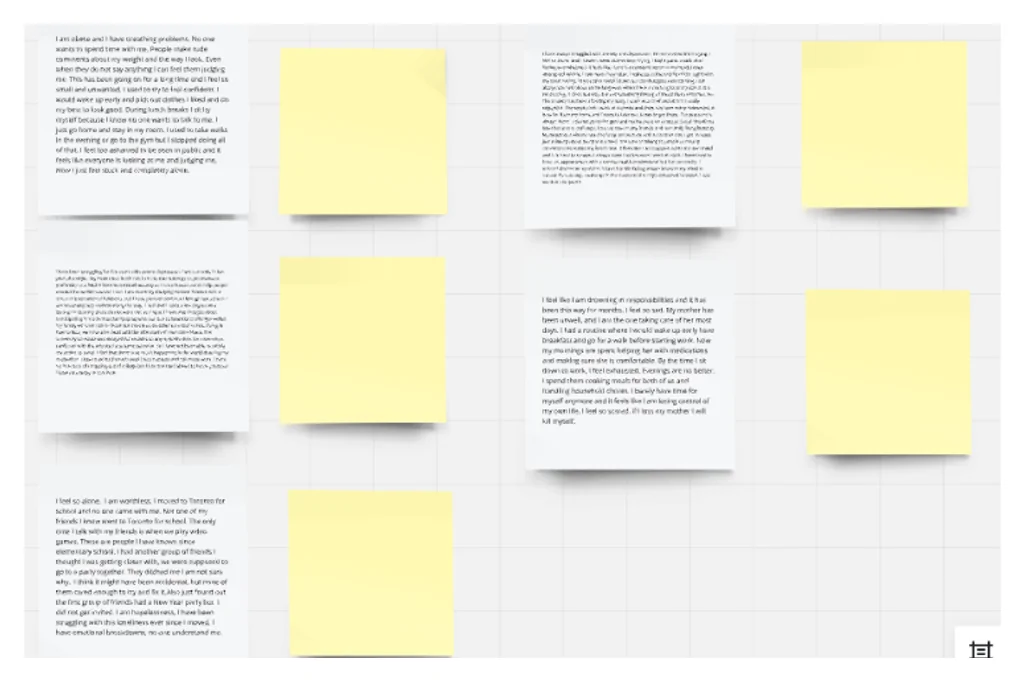
Practitioners annotating depression symptoms from the posts on the Miro platform during co-design workshops, comparing their own clinical assessments with DepressionSymp model outputs.

#### Ethical Considerations

This study received approval from the Lancaster University Institutional Ethics Committee (ID 3530). Written informed consent was obtained from all participants via a signed consent form, completed prior to the start of the study, covering audio recording, transcription, and anonymized use of their data for research; all data were anonymized and deidentified prior to analysis. Participants received a £40 (US $54) Amazon voucher as compensation.

#### Data Analysis

The workshops’ audio recordings were fully transcribed and analyzed using ATLAS.ti software (ATLAS.ti Scientific Software Development GmbH) [74]. This study used Swain’s hybrid thematic analysis approach, combining deductive and inductive coding and theme development [75]. By using Swain’s approach, we treated codes and themes as equivalent rather than distinct hierarchical levels. Deductive codes were informed by AI ethics literature on accuracy, reliability, interpretability, and trust [47,48], as well as ethical challenges [13,76]; these codes were defined in advance, based on existing theory rather than the workshop data itself. Inductive codes emerged directly from workshop data, particularly recommendations for improvement, meaning they were not decided beforehand but developed from what practitioners actually said. Coding followed a structured, iterative process involving ongoing discussion within the research team, drawing on complementary expertise in AI-HCI, mental health, and ethics. The first author conducted an initial coding pass on a subset of the workshop data, generating a preliminary set of codes; the second author then independently reviewed these codes, and the 2 discussed their definitions, distinctiveness, and overall organization before finalizing the codebook. This iterative discussion process, involving independent coding and review by a second author with expertise in HCI and mental health, served as an investigator triangulation strategy to enhance the trustworthiness and rigor of the coding and theme development process. Following the Swain approach, the codes generated during coding were organized and grouped into a smaller set of main codes (Swain phase 3, stage 2). Accuracy and reliability were merged into one main code, as were interpretability and trust, since each pair was closely related. Ethical challenges and recommendations for improvements were retained as separate main codes, as they captured distinct content that cut across the 4 ethical principles rather than mapping onto any single one. This process resulted in the 4 main codes reported in Table 3. Reporting code frequencies is consistent with this hybrid thematic analysis approach. The final main codes and their frequencies are summarized in Table 3.

**Table 3.** Main codes and their frequencies identified through qualitative analysis of workshop data.

Codes	Frequencies
Accuracy and reliability	198
Interpretability and trust	40
Ethical challenges	51
Recommendations for improvements	91

### Results

#### Overview

Our findings are organized around 4 AI ethical principles: accuracy and reliability, interpretability, and trust [47,48], assessed through practitioners’ hands-on interaction with the model (Figure 4). Accuracy and reliability reflect the model’s ability to correctly and consistently extract symptoms compared with practitioners’ assessments. Interpretability and trust capture whether practitioners could understand, navigate, and feel confident in using the model’s outputs. Other principles [13], such as fairness and privacy, were beyond this study’s scope. Ethical challenges capture broader concerns about the limitations and risks of applying the model in clinical practice, including missing contextual information, cultural and linguistic differences, and the need for human oversight. Recommendations for improvements capture practitioners’ proposed changes to the model and its assessment process.

**Figure 4.**
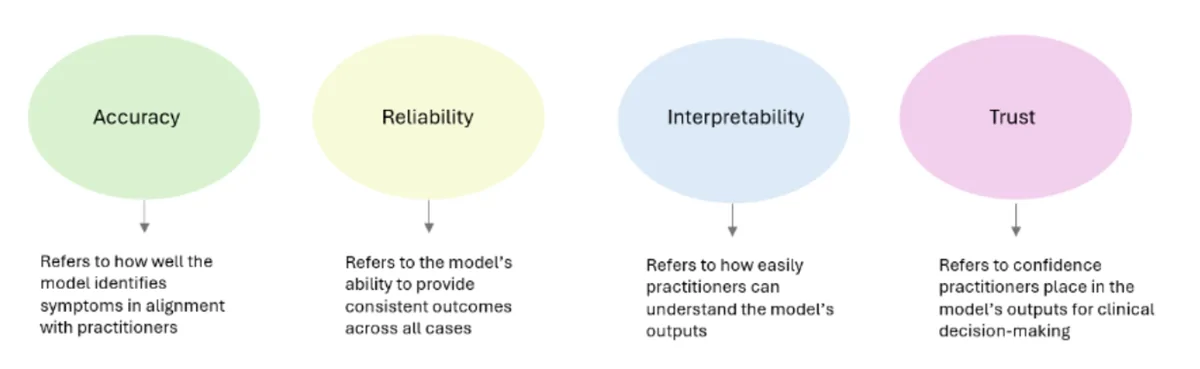
Description of the AI ethical principles used to frame DepressionSymp.

#### Accuracy and Reliability

Our findings showed that DepressionSymp’s identification of symptoms was closely aligned with practitioners’ judgments, with only minor nuances in how categories were applied (Figure 5). Participants (P1-P6) consistently noted that the model effectively captured core emotions, thoughts, and negative feelings, reflecting their own assessments. However, our findings also indicate that certain indirect symptoms were classified under lifestyle by the model, whereas practitioners considered them more appropriately placed under symptoms.

**Figure 5.**
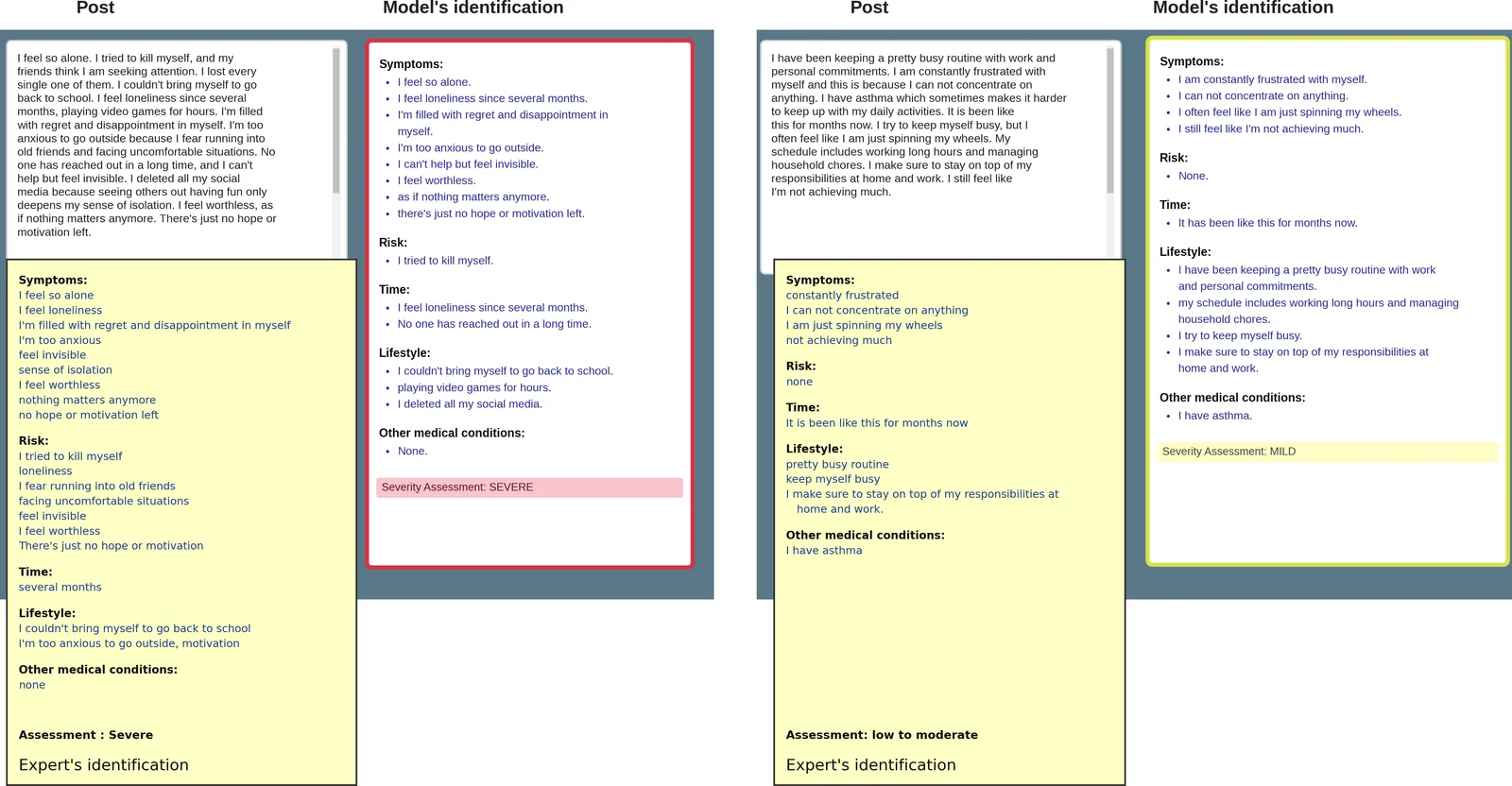
Comparison between practitioners’ manual symptom identification and DepressionSymp 1.0 model outputs across 5 posts.

A significant finding was the role of repetition. In clinical practice, repeated expressions of hopelessness are often interpreted as indicators of greater severity (P1 and P2). As one practitioner stated:

When I’m with a client, if they say repeatedly but in slightly different phrases, ‘I feel hopeless. I feel fed up.’... the amount of times that they express something of a similar theme [DepressionSymp] gives an indication to how severe those symptoms are*.*

[P1]

Another practitioner noted that DepressionSymp sometimes consolidated repeated phrases into a single entry, considered appropriate: “[DepressionSymp] included one line that I didn’t - just because it was repeated” (P3). Participants’ identification of time-related factors, including duration, onset, and fluctuations, consistently matched the model’s, demonstrating clear alignment in how time was treated as a relevant evaluative factor (P1-P6).

All participants (P1-P6) identified medical conditions in alignment with the model, though physical symptoms such as fatigue or body pain may overlap with depression (P4 and P5), and some conditions not directly linked to mental health could still increase risk depending on the case (P5). Our findings reflected differences between human and DepressionSymp performance, with the model performing consistently across the 5 posts provided to each participant, while practitioners’ own assessments varied. P5, for instance, acknowledged missing more symptoms than the model. Several practitioners (P2, P3, and P4) also noted that DepressionSymp identified symptoms they had initially missed, and agreed on review that these were valid, though this reflects the specific posts reviewed rather than a general claim about the model’s reliability across broader clinical contexts.

#### Interpretability and Trust

Our findings suggest that participants generally viewed the model’s outputs as reasonable and aligned with their expectations, contributing to their confidence in the model (P1-P6): “It made sense that the things that it picked I felt were reasonable” (P1). Similarly, participants indicated that they could understand the categories used by the model and why these categories were applied:

I think when I was going through it and then I see that the model and what the model came up with.. I could understand… but I can understand why the model has used those categories.

[P4]

Findings show that participants (P3, P4, and P5) consistently highlighted the model’s intuitive design and described it as straightforward to understand without requiring additional explanation (P4). Outputs were regarded as clear and reassuring, contributing to participants’ confidence in using the model (P5).

A key strength identified was the model’s ability to simplify complex information into accessible formats (list) (P1, P2):

I guess at the current moment it gives a general overview of the symptoms and the lifestyle of the person. So if we’re ignoring the risk factor at the minute it gives it simplifies the text into bullet points that you can scan.

[P1]

This simplification was also recognized as particularly valuable for supporting health practitioners with specific needs, such as dyslexia:

so that might be helpful to health practitioners that might have like dyslexia or something like it could potentially help them to scan the information… to view any other medical needs, i think it’s good for an overview to simplify.

[P1]

Our findings indicate that the model shows strong potential to support clinical assessment and practitioners’ decision-making. Participants highlighted its usefulness in helping practitioners determine whether to take on a client and how to prioritize cases:

I feel like it would become more trustworthy with the adjustments that we’ve discussed. And then potentially places even in private practice might use it, for example, with counselling directory to help practitioners decide whether or not to take on a client, or how to prioritize them.

[P1]

Another key strength was the model’s explainability, which allowed for the identification and clustering of individual struggles to inform personalized interventions:

it could be extremely easily fed into case formulations… and in case formulations, then we can extract the interventions… So if we can see how the person struggles and we have a cluster and then we say when this cluster comes, we do that.

[P6]

Participants also praised the clarity of the outputs and how they facilitated conversations with clients (P2 and P6). The bullet-pointed, summarized format was seen as well aligned with conversational practice, supporting clinicians in engaging with patients and addressing their concerns (P2), and outputs were considered transparent, with the model’s reasoning clear even when participants’ own interpretations sometimes differed (P6). Participants also suggested that visual cues, such as color coding, could further enhance interpretability. For instance, highlighting main words contributing to severity assessments (eg, references to suicide attempts) in red color could make it clearer why the model classified certain inputs as severe (P6).

#### Ethical Challenges

Findings indicate the model’s limited ability to process nonverbal elements such as speech rate, tone, and inflection (P3), as well as visual and behavioral cues such as appearance, hygiene, and body language (P1, P2, and P3), which can reveal underlying issues even when clients appear outwardly well:

All of those kinds of things... their appearance and the way they smell... it does help to have that visual input... Sometimes people can be very well put together and still be actively suicidal, but it does help to have that visual input*.*

[P1]

The model may also struggle to identify suicidal tendencies without this context (P1), and may miss nuanced information that is not explicitly stated: *“*this teen is extremely lonely and feeling very low self-worth, but they never used any of those words*”* (P3). While useful for routine assessments, it is less suited to complex cases requiring nuanced human understanding: *“*if we had a more complicated, complex case of an individual... that would still be better suited to a human researcher*”* (P4).

Practitioners agreed with the model’s conclusions in many cases, though differences in understanding risk, lifestyle factors, and cultural nuance could still arise: “there could be a risk of discrimination... I’m not sure if our model can become more culturally competent to pick up on cultural nuances” (P4).

Ultimately, practitioners emphasized that the model must prioritize understanding the broader humanity of clients rather than reducing them to symptoms, risks, and severity (P6).

The *DSM-5* provides a severity spectrum for depression (mild, moderate, and severe) without specifying the exact number of symptoms required for each level [63]. For consistency, we therefore operationalized severity using symptom counts: mild corresponding to 5 symptoms (the diagnostic minimum), moderate to 6 or 7, and severe to more than 7. Practitioners’ approaches to severity diverged from this model, drawing instead on descriptive language, contextual factors, and perceived risk rather than quantitative thresholds. Severity was sometimes linked to specific language, such as words like *“*dark” or *“*heavy” (P5), or to the immediacy of risk, such as suicidal thoughts, which practitioners considered critical regardless of symptom count (P2 and P4). Several noted that even 4 or 5 symptoms combined with markers of loneliness could indicate a severe case (P1). Practitioners also emphasized the subjective, fluctuating nature of severity. For instance, fleeting suicidal thoughts did not always indicate high risk (P2 and P3), and quantitative scoring systems had proven unreliable in serious cases (P3). Other considerations included history of risky behaviors, social support, and motivation for the future (P3); caution in cases of suicidal thoughts even without a concrete plan (P4); the impact of depression on lifestyle and physical health even without suicidal ideation (P5); and the extent to which symptoms disrupted daily living or reflected past trauma (P6).

While DepressionSymp 1.0 and practitioners agreed on a large proportion of extracted symptoms, discrepancies remained across all 6 practitioners, with symptoms not classified by the model under ”symptoms” instead allocated to lifestyle or risk categories. Practitioners consistently regarded these as indirect symptoms embedded in those contexts rather than as missed items. For example, the phrase ”lack of skills to talk to others” was classified by the model as a symptom, whereas a practitioner considered it potentially indicative of a medical condition (P4). More broadly, factors such as disengagement with health activities, major life changes, or academic and financial struggles did not explicitly express emotional distress, yet practitioners understood them as meaningful indicators of underlying symptoms, whereas DepressionSymp 1.0 tended to classify them more narrowly as lifestyle. This points to a limitation in contextual sensitivity, and also shows that discrepancies between model classification and practitioner judgment are not simply a matter of the model being wrong, but reflect genuine interpretive differences in how such experiences are understood.

Although some participants noted that certain lifestyle events could be considered risks depending on the case (P1, P2, and P4), P1 believed some lifestyle aspects should also be treated as risk indicators:

lifestyle is almost on par with symptoms, but sometimes doesn’t quite have the same things... I think that’s more because there’s some things in lifestyle that I would sometimes put in risk.

[P1]

Certain occupations, such as being a retail manager, were seen as lifestyle factors that could also signal stress and risk (P2), and missing assignments were viewed as both a lifestyle factor and a symptom, potentially linked to conditions like attention-deficit/hyperactivity disorder (ADHD; P2). Weather was similarly seen as either a lifestyle factor or a symptom depending on its perceived impact (P3).

Other lifestyle factors could signal risk more directly: the absence of effective therapy, for instance, was seen as raising risk if a client remained unhelped despite seeking support (P5): *“*is a therapy working?... they’re not sort of taking anything good from it, and they don’t have a plan for... There’s more risk as well*”* (P5). Emotive language also blurred the lines between symptoms and lifestyle, with severity assessed differently depending on perspective (P6):

the differences have been around emotive language and identifying in which category that emotive language goes... those two would get mixed up*.*

[P6]

Findings also focused on the overlap between symptoms and risks, particularly in relation to suicidal thoughts. Practitioners (P1 and P2) duplicated certain risks within the symptoms, explaining that keywords such as ”suicide” should appear in both categories: *“*it’s good that it’s capturing suicide under the risk... but I believe suicide needs to come under a symptom as well*”* (P2).

This illustrates that the boundaries between symptoms and risks are not always distinct, and that DepressionSymp requires a more careful approach to such cases (P1). While DepressionSymp generally aligned with practitioner assessments, these occasional differences in classifying symptoms versus risks point to a limitation worth addressing in future refinement.

Both DepressionSymp and participants consistently identified immediate or urgent risks as critical, though DepressionSymp primarily focused on suicidal ideation while participants (P1, P2, P4, and P6) described a wider range of factors, viewing risk as a gradually developing spectrum rather than always indicating immediate life threat (P4 and P6). Each practitioner highlighted different considerations. Lifestyle and situational stressors, including unemployment, work stress, academic pressure, and chronic stress, were seen as risks: *“*unemployment increases the risk” (P2). Social and emotional indicators of risk, including social withdrawal, hopelessness, financial stress, and family-related stress, were also important signals, with isolation noted as a particularly strong indicator of suicidality (P1) and family loss described as escalating vulnerability (P1). Technology-driven stressors were also raised, with social media use seen as capable of fueling vulnerability to self-harm, and excessive scrolling described as both a potential risk and a symptom (P4). Physical and behavioral changes, such as visible tension or aimless pacing, and changes in how people spend their time, such as reduced family engagement, were seen as potential risk indicators (P4). Finally, the duration of a mental health issue was seen as a factor in assessing risk, with longer periods of distress potentially increasing severity and worsening outcomes (P4).

Our findings highlight several important ethical challenges in how the model may be applied in practice. One concern related to bias in interpretation, with participants noting the risk of perpetuating stereotypes that could retraumatize or victimize clients (P1 and P4). Another challenge involved nuanced communication styles: clients may use abstract or poetic expressions requiring sensitive interpretation (P5), or initially provide limited information, requiring the model to adapt and prompt meaningful responses (P4). Individuals may use defense mechanisms, such as humor or superficial language, to describe experiences of severe trauma, which the model may fail to recognize: *“*someone can tell you things that are very severe in a very happy way. they can even joke about it” (P6). This disconnection is a challenge for our model; if clients input incorrect or incomplete data, it can impact the accuracy of the assessment and severity determination (P1).

#### Recommendations to Improve DepressionSymp 1.0

Practitioners proposed reclassifying certain lifestyle indicators into a more detailed symptoms category, comprising: (1) emotional distress (feelings and thoughts); (2) disengagement from health activities: stopping previously routine or enjoyable activities (such as hobbies, exercise, or daily self-care); (3) disengagement from social interaction: disconnection from relationships or reduced social engagement; (4) financial instability: difficulties in managing finances (eg, limited income, debt, and rising costs of living); (5) sudden life transitions or changes: abrupt shifts in living arrangements, careers, or personal relationships; (6) generalization and social perception (fear of judgment): use of absolute terms or expressions of fear of judgment; (7) problematic behavior: initiation or return to unhealthy coping mechanisms (eg, smoking and alcohol use); and (8) time management challenges: difficulties in organizing responsibilities, meeting deadlines, or managing daily tasks.

To support more comprehensive assessment, practitioners recommended prompts addressing 5 areas: daily tasks and self-care, exploring how routines affect energy and motivation, not just completion; care plans and safety mechanisms, covering protective factors, safety planning, and support systems; coping mechanisms and protective factors, exploring current strategies, resilience, and existing supports; history and context, gathering childhood history, treatment history, and demographic information such as age; and risk factors, addressing external risks such as weather, social isolation, or verbal abuse, along with severity-based assessment of self-harm risk.

These recommendations targeted 3 ethical principles: interpretability, to reduce misclassification between lifestyle, symptoms, and risk categories; accuracy and reliability, to recognize behavioral and situational indicators as meaningful symptoms or risk; and trust, to ensure the model understands subtle or indirect language and context.

## Study 2

### Methods

#### Overview

The insights from the co-design workshops were next used to inform the revised design of the DepressionSymp model. This section describes the development of DepressionSymp 2.0.

#### Data Preparation

For model 2.0, we shifted from fine-tuning to few-shot learning, motivated by 2 factors: the LIWC tool did not fully address the newer, nuanced categories identified by practitioners, and the limited size of the revised dataset (30 annotated posts across 10 categories) made fine-tuning insufficient, whereas few-shot learning is designed to generalize effectively from small samples [77,78]. The lead researcher implemented these practitioner-proposed categories and re-annotated the sample into 10 revised categories: risk, emotional distress, disengagement from health activities, disengagement from social interaction, financial instability, fear of judgment, life transitions and changes, problematic behavior, time-management challenges, and other medical conditions.

These expanded categories, informed by practitioners’ feedback, also align with established clinical frameworks: the *DSM-5* states that depression extends beyond core emotional symptoms to include behavioral, cognitive, and psychosocial dimensions [63], with disengagement from health and social activities mapping onto diminished interest or pleasure in activities, fear of judgment and generalization reflecting cognitive distortions, financial instability and life transitions representing recognized psychosocial stressors, problematic behavior such as substance use reflecting a well-established comorbidity and coping response, and time management challenges reflecting executive functioning difficulties. This deeper categorization supports more targeted, personalized interventions while improving the transparency and face validity of model outputs.

#### Model Development: DepressionSymp 2.0

The model was evaluated on these posts using zero-shot and few-shot learning, analyzing posts without example prompts in the zero-shot setting [79], then incrementally with 6, 12, and 17-shot examples [80], allowing it to progressively learn from task-specific examples and improve symptom classification and information extraction (Figure 6).

**Figure 6.**
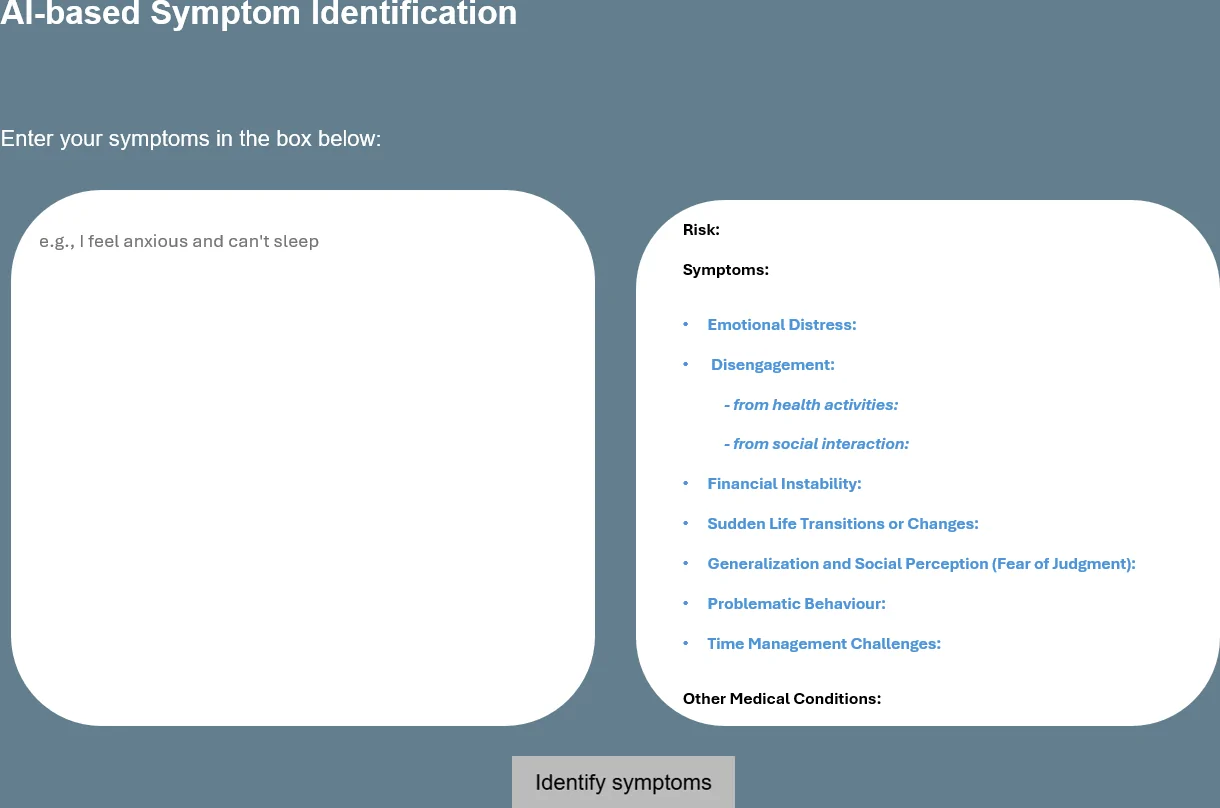
DepressionSymp 2.0 model showing (left) a user’s input post describing mental health experiences, and (right) the model’s structured output.

#### Model Testing

The few-shot learning approach was applied to GPT-3 based on practitioners’ feedback, with performance improving substantially from zero-shot to few-shot learning, then showing progressively smaller gains as example counts increased from 6- to 17-shot, indicating the model was approaching saturation (Table 4).

**Table 4.** ROUGE^a^ -1/2/L accuracy for zero-shot versus 6, 12, and 17-shot learning, with improvement at each step.

ROUGE	Zero-shot	6-shot	Improvement	12-shot	Improvement	17-shot	Improvement
ROUGE-1	0.5335	0.8267	29.3%	0.8454	2%	0.8486	0.32%
ROUGE-2	0.3944	0.7325	33.8%	0.7626	3%	0.7715	1%
ROUGE-L	0.4355	0.6630	22.8%	0.6841	2%	0.6942	1%
Test examples	30 examples	24 examples	—^b^	18 examples	—	13 examples	—

a ROUGE: recall-oriented understudy for gisting evaluation.

b Not available.

#### Participants

For study 2, we also used a purposive small sample size including 8 practitioners. This remained within the range associated with data sufficiency for homogeneous populations pursuing a narrow research objective [72], while providing a modest increase in information power by recruiting a somewhat more experienced group (3‐13 years of professional experience, vs 2‐10 years in study 1) to evaluate the revised, more detailed ten-category model [73]. A larger sample also reflected that DepressionSymp 2.0 introduced substantially more categories than the previous version 1.0, thus benefiting from feedback from additional practitioner perspectives to evaluate whether the expanded categorization held up in practice. To evaluate DepressionSymp 2.0, we recruited 8 mental health practitioners (5 female and 3 male), aged 27‐47, with 3‐13 years of experience across private and public mental health practices, including university counseling services. Full demographic and professional details are provided in Multimedia Appendix 1. Workshops for study 2 were conducted in August 2025.

#### Workshop Procedure

Study 2 followed the same procedure as study 1, with 2 differences: DepressionSymp 2.0 was evaluated instead of 1.0, and participants reviewed 5 new posts not used in study 1 (since those posts had been included in the model’s few-shot training). In total, 8 participants evaluated 40 unseen posts. Study 2 followed the same ethical procedures as study 1.

#### Data Analysis

As in study 1, workshop recordings were transcribed and analyzed using ATLAS.ti software [74] with the same coding scheme (Table 5).

**Table 5.** Main codes and their frequencies identified through qualitative analysis of workshop data.

Codes	Frequencies
Accuracy and reliability	95
Interpretability and trust	84
Ethical challenges	65
Recommendations for improvements	42

### Results

#### Accuracy and Reliability

DepressionSymp 2.0 achieved strong alignment with practitioners’ assessments, with many (P1-P8) estimating similarity rates around 80%‐95%, and outputs were consistently described as accurate and closely matched to practitioners’ judgments (P1, P2, and P5), with only minor differences in interpretation (P4). Practitioners noted that the system consistently captured key clinical content across posts (P3, P6, P7, and P8), occasionally identifying symptoms they had overlooked, such as emotional distress (P1 and P4), and its ability to classify depression into multiple components was seen as enhancing clinical use by helping isolate distinct contributors to a client’s difficulties (P3, P7, and P8). However, challenges remained, mostly regarding risk identification. Some practitioners noted that 2.0 occasionally classified statements as risk that they interpreted differently, such as medication use rather than self-harm (P1), and others questioned how the system distinguished crisis indicators from lower-level risks (P3). Some experiences also fit multiple categories: financial stressors were viewed as either symptom or risk depending on interpretation (P1 and P3), and participants noted differences in how problematic behavior was separated from disengagement, and fear of judgment from emotional distress, reflecting practitioners’ reliance on clinical expertise to integrate contextual meaning that the model tended to separate into distinct categories.

#### Interpretability and Trust

A consistent improvement in 2.0 was the clarity of categories. Practitioners stated that the system’s allocations felt coherent and intuitive, and most could understand the underlying reasoning without additional explanation (P1, P2, P4, P5, P7, P8, and P8):

if we took risk out of it completely, I’d be able to trust it on the basis that it was saying… these are some symptoms, this is what’s going on for this person [... ] I could get like a good overview of what is going on for the person … but when it comes to risk, obviously that’s like a massive thing for mental health ... if you took risk, I think it’s pretty trustworthy... these are the things that the person is struggling with… If I got that, I could get a good representation... AB and C and this is what you’ve been struggling with.

[P1]

Another example was that elements such as financial strain or disengagement from activities were presented in a way that practitioners found clear and understandable. While some practitioners suggested that certain categories could be broadened for greater flexibility (eg, disengagement not only from health activities but from activities in general), the overall impression was that 2.0 provided a transparent set of outputs.

An added value of the perceived transparency of the 2.0 model was its potential to inform intervention planning. Practitioners mentioned that the structured categories offered a clearer starting point for interventions, keeping them focused on specific criteria to address (P3):

if a client’s really struggling with social stuff and they don’t have any friends or whatever, but as part of the treatment, you can suggest that they join a group or they speak to someone… It also keeps you focused as a therapist as well because you feel like you have these, like kind of criteria... that you want to address.

[P3]

By flagging areas such as social isolation, the model helped practitioners translate insights into actionable steps, such as encouraging clients to join groups, an improvement over model 1.0, where outputs were mainly valued for decision support (P3 and P5). The structured categories also helped clarify client issues and highlight key information, allowing practitioners to identify next steps such as addressing social interaction needs or time management challenges (P7 and P8):

quite useful so that the mental health practitioners would know that there are things to play on... things to work on... there’s hope.. there’s something that we could encourage... or sorted into a priority the decision would be to act quickly.

[P7]

A further point supporting trust came from model 2.0’s reliance on client language. Practitioners appreciated that the system categorized inputs directly from the words provided by clients rather than making assumptions (P7 and P5), viewing this as a safeguard against bias that kept outputs close to clients’ lived experiences and provided a clear, transparent representation of what clients expressed (P3):

you need to know how severe it is... so it’s so important that it captures exactly what is being said and doesn’t change that... it’s better that it takes a client language.. because that way we’re not making any assumption it’s coming from exactly what the clients put*.*

[P3]

Another key element of trust stemmed from the model’s potential to support preassessment: AI-generated summaries before meeting a client would shorten initial sessions, support note-taking and early formulation, and reduce repeated questioning, freeing more time for therapeutic engagement (P1, P5, and P6), while online submission of client self-reports further strengthened this by capturing information before the session began (P1, P4, and P8):

for a counseling or psychotherapy service manager who’s got to assign.. lots of clients that come in and assign them to appropriate practitioners, and when I say appropriate, appropriate in terms of what they specialized in... AI can basically filter out anyone who has suicide risks, and these people shouldn’t be given to trainees because it’s not really fair... It’s not really safe... so in that way it could really help in allocating clients*.*

[P4]

Trust was also supported by the model’s clear categorization and structure, which allowed practitioners to navigate data without scanning the entire record (P5). This structured overview provided a succinct yet informative representation of the client’s life circumstances, such as financial or social difficulties, which practitioners appreciated (P2, P5, P6, and P8).

#### Ethical Challenges

Findings reveal that text-only analysis misses the *“*meaning between the words,” as therapeutic understanding extends beyond text to tone, cadence, and clinical judgment (P2); practitioners noted that *“*reading between the lines” often reveals risks not explicitly expressed, meaning models relying only on literal text may overlook subtle cues (P4). They also highlighted the difficulty of detecting subtle depression, denial, and defensive minimization, as clients may express symptoms indirectly, conceal their severity, or use indirect, poetic language that indicates suicidality without explicit terms. Automation without human oversight was seen as a safeguarding risk, as fully automated allocation could route high-risk clients to undertrained practitioners (P4). Model reliability is also constrained by honesty, disclosure, and stigma, as clients may omit or minimize sensitive information; systems should therefore anticipate ambiguity rather than assume complete disclosure (P4). Practitioners further emphasized that holistic risk assessment should include social context, such as the absence of close family or friends, which may substantially increase risk (P5).

Participants also noted that the model’s outputs are adequate for brief screenings but insufficient for deeper clinical formulation without additional context (P5), and that real-time deployment is challenging without guided prompts, since clinicians typically ask far more in a session than a model currently anticipates (P5). AI cannot capture what is not said, while humans often can, as clinicians detect unspoken affect and subtext that sole reliance on text risks missing (P6); client review and correction loops, where clients can review, agree with, or dispute symptom summaries before automated outputs inform decisions, may help address this (P6). Language and culture also interact (P6, P7, and P8), with words carrying different implications depending on background; while the model can categorize information consistently, practitioners saw it as their responsibility to remain sensitive to these nuances for each client.

#### Recommendations to Improve DepressionSymp 2.0

Practitioners proposed seven types of risk to guide a more detailed risk evaluation layer: (1) different levels of risk: the system should distinguish between thoughts, plans, and immediate intent; (2) risk to self and risk to others: risks may involve self-harm, exploitation, or potential harm to others, and should be clearly separated; (3) crisis vs ongoing risk: a dedicated crisis category should capture immediate threats, while ongoing/general risk addresses chronic conditions such as long-term depression; (4) historic vs current risk: the system should enable assessment of both past risk incidents and present; (5) risk factors and protective factors: broader social and behavioral elements such as substance use, living alone, and barriers to treatment should be integrated alongside protective factors such as family support, employment, or resilience; (6) transparency in risk categorization: the system should explain why risk was flagged, providing the original sentence with explanation; and (7) traffic light system: a green-amber-red framework should be adopted to visualize levels of severity.

## Discussion

### Principal Findings and Comparison With Prior Work

This study explored how iterative co-design with mental health practitioners shaped the development of DepressionSymp, an LLM-based model for identifying depression symptoms. Findings showed that practitioners interpreted client narratives using contextual judgment not fully captured by the initial model. Experiences such as social withdrawal, financial difficulties, and major life changes were recognized as indirect expressions of depression rather than lifestyle factors, informing a more clinically relevant representation of symptoms that improved output interpretability and supported preassessment and intervention planning. Evaluating the model through the ethical principles of accuracy, reliability, interpretability, and trust identified strengths, limitations, and priorities for refinement throughout the co-design process. Across both studies, practitioners consistently identified risk assessment as the most ethically sensitive aspect of the model, which should remain under human clinical judgment.

Regarding RQ1, DepressionSymp 1.0 aligned closely with reference annotations, achieving ROUGE-1 (91%), ROUGE-2 (88%), and ROUGE-L (87%) *F*_1_-scores, comparing positively to prior summarization efforts such as BARTlarge-CNN (ROUGE-1: 0.810) and Pegasus-large (ROUGE-1: 0.829) in simulated mental health dialogues [34,81]. DepressionSymp 2.0 demonstrated reasonable accuracy using few-shot learning, improving significantly from zero-shot to 6-shot examples (ROUGE-1: 0.5335 to 0.8267,+22.8%). Practitioners valued the multicategory structured outputs over binary classifications [35,38,82,83], finding them easier to interpret and review. ROUGE was selected because the task requires preserving exact client wording, where terms such as ”hopeless” carry diagnostic meaning that semantic metrics such as BERTScore may obscure, since BERTScore also ignores word order [84,85]; bilingual evaluation understudy (BLEU) was not used as it measures n-gram precision only and applies an unsuitable brevity penalty [86]. Practitioners perceived DepressionSymp 2.0 more favorably than 1.0 due to its greater interpretability. Unlike LLAMADRS [87] (numerical Montgomery-Åsberg Depression Rating Scale [MADRS] scores) and Qwen2-72B-Instruct [88] (binary symptom judgments), DepressionSymp preserves client language and captures indirect symptom indicators, extending LLM-based approaches to underexplored symptom categories. Future work with larger, more diverse datasets could improve identification of complex language patterns less common in the current training data [89].

Regarding RQ2, practitioners in study 1 indicated that lifestyle factors such as social withdrawal and activity changes function as indirect symptoms of depression [90,91], leading DepressionSymp 2.0 to expand from 5 to 10 symptom categories. Study 2 confirmed improved alignment, with practitioners noting that 2.0 consistently captured key symptoms, including some previously overlooked. Where study 1 revealed interpretive disagreement over whether items were symptoms, lifestyle factors, or risk indicators [92], 2.0’s clearer, consensus-based definitions were reported as coherent and intuitive without additional explanation, demonstrating improved reliability [93], though risk categorization remained the most challenging area. This aligns with prior work on structured output reducing cognitive load and improving reliability in mental health contexts [93], extended here by showing that practitioners similarly valued structured categories for presenting complex client information clearly, while our approach preserves client language and captures both direct and indirect symptoms for richer, more clinically nuanced outputs.

Practitioners also proposed structured prompting questions (eg, daily functioning, care plans, coping mechanisms, and risk factors) to support more comprehensive assessment [94]. DepressionSymp 2.0 addressed several limitations through: (1) clearer categorical reasoning without additional explanation; (2) use of clients’ own language against interpretive bias; (3) structured outputs supporting intervention planning; and (4) a preassessment role organizing information before sessions, reducing repetitive questions. This addresses recent calls for transparency, reliability, and human oversight in ethical LLM use in mental health [95], achieved by co-designing symptom categories with practitioners to improve consistency and interpretability. In contrast to studies showing LLMs often produce inconsistent responses that undermine trust and increase clinical-error risk [95], our approach focuses on output transparency and detailed symptom categorization rather than broad summaries [22,68], retaining features such as sentence frequency, exact client language, and intensity of expression, each reflecting clinically important differences in symptom severity [96].

Study 2 showed stronger trust overall, with practitioners reporting “strong confidence in symptom identification” while maintaining “conditional trust” in risk assessment, highlighting the need for clearer reasoning and follow-up questions to support risk interpretation. However, concerns about risk categorization and cultural expression pointed to broader ethical dimensions, such as fairness and accountability, that these 4 criteria could not fully capture [97], suggesting future work should extend the evaluation framework as DepressionSymp moves toward clinical deployment. In response, practitioners proposed a seven-layer risk framework: (1) different levels of risk (thoughts, plans, immediate intent), (2) risk to self versus others, (3) crisis versus ongoing risk, (4) historic versus current risk, (5) risk and protective factors, (6) transparency in risk categorization, and (7) a traffic light system.

Regarding RQ3, the iterative design of the DepressionSymp models identified key ethical considerations for integrating LLMs into mental health symptom identification. Prior work has highlighted challenges related to clinical trust, bias, and reliability [12,88], and existing tools often rely on fixed question formats or unclear outputs that limit clinicians’ interpretation of assessments [38]. Our findings show that symptom categories overlap in practice and that LLMs require contextual sensitivity when symptom meaning and cultural context extend beyond the text.

Another significant ethical challenge, consistent with prior work [98], is that LLMs lack the ability to process nonverbal cues, such as tone or body language, while language and cultural differences may also affect how symptoms or risk are expressed and interpreted, meaning models trained primarily on textual patterns may not fully capture culturally nuanced experiences [21]. For instance, a statement like “I just want peace” may indicate substantial risk depending on context but could be interpreted neutrally by the AI, and defensive or poetic expressions may similarly obscure symptom severity. Integrating nonverbal cues with textual analysis, for example by combining LLMs with multimodal AI systems that analyze video and facial expressions alongside text [98-100], could support practitioners in detecting risk and nuance, offering a more comprehensive understanding of an individual’s mental state.

Our work also highlights an ethical challenge in holistic risk assessment. While prior work has focused on immediate risk based on symptom measures [101-103], our findings show that severity also depends on contextual, historical, and social factors, such as social isolation, family loss, and chronic stress, making symptom-count approaches prone to oversimplifying complex experiences. These limitations carry ethical consequences beyond technical performance, as both false negatives and false positives have important implications in mental health [104]: failing to identify hopelessness, suicidal ideation, or related indicators such as social withdrawal, disengagement, or financial instability results in an incomplete symptom profile that may misdirect intervention and increase patient risk [105], while assigning irrelevant sentences to incorrect categories increases practitioners’ cognitive load and may lead to interventions that do not reflect clients’ needs [106]. These errors differ in ethical severity: missing suicidality poses the greatest risk to patient safety, while symptom/lifestyle misclassification carries lower immediate consequences. Model errors should therefore be evaluated by clinical impact, with failure-case analysis prioritized for high-risk categories like suicidality before deployment.

### Implications for Design: LLM Features

Our findings informed 3 specific design implications for supporting identification of depression symptoms.

#### Supporting Contextual Understanding of Symptoms and Overall Severity

Study outcomes confirm the value of our categories to support the identification of depression symptoms, including more detailed and subtle expressions of emotions and thoughts. Longer or more diverse posts are also likely to contain a broader range of depression symptoms [107]. To capture broader context and measure overall severity levels, we suggest that while shorter texts may present clearer and more direct symptoms, they often provide limited context for assessing symptoms’ severity. In contrast, increasing the length of input text allows for a more in-depth analysis of multiple emotional expressions within each post [28]. Overall, we call for considering training LLMs with different lengths of text to enable the model to capture both immediate indicators of distress and deeper contextual patterns.

#### Supporting Culturally Grounded Lexicons and Adaptive Interpretation Mechanisms for Symptom Identification

Our findings confirm the need for LLM systems that support cultural adaptability, since meaning, tone, and pragmatics vary significantly across cultural backgrounds even within English [108]. This requires curated multilingual corpora and datasets of culturally marked expressions [103,109,110], alongside personalized, context-specific interpretation that distinguishes figurative from clinically high-risk expressions [111,112]. Specifically, we propose a culture-conditioned mapping layer applied after LLM-based categorization, similar to the *DSM-5* cultural formulation framework, acting as an intermediate reasoning step that aligns detected symptoms with culture-specific meanings, idioms of distress, and explanatory models of illness [113,114] – distinguishing expressions such as ”my chest is tight” from biomedical symptoms.

#### Supporting Detailed Risk Evaluation

Our findings propose integrating a detailed risk evaluation layer [115,116] that assesses the level and type of risk in each classification, including indirectly expressed risks, such as repeated mentions of isolation signaling heightened vulnerability over time, applying transparent criteria including frequency, severity markers, and contextual descriptors of intensity, with words such as ”very” or ”really” weighted more heavily [38,117]. Our recommended 7 types of risk offer a useful starting vocabulary: different levels of risk (thoughts, plans, and immediate risk), risk to self versus others, crisis versus ongoing risk, historic versus current risk, risk and protective factors, transparency in risk categorization, and a traffic light system. Risks can also be grouped under each symptom category as direct (eg, explicit suicidal ideation) or indirect (eg, persistent withdrawal, fear of judgment, and reduced engagement in daily life) [118]; consolidating and weighting these features could help the model generate layered risk profiles capturing both immediate and emerging vulnerabilities, moving beyond surface-level detection. We further recommend presenting results through visual tools such as category-level dashboards [119] and risk explanations [35], strengthening transparency by making the model’s reasoning visible and actionable.

### Implications for Design Research: Practitioners-in-the-Loop

Despite improvements, LLM-based systems may still produce inconsistent, biased, or inaccurate outputs. We recommend embedding a human-in-the-loop mechanism in DepressionSymp [120], where practitioners review and correct preliminary LLM-generated assessments, flag high-risk cases, and provide feedback that iteratively improves reliability [121]. We further argue for extending this into staggered co-design and cross-validation with expert practitioners.

#### Engaging Mental Practitioners in Iterative, Staggered Co-Design and Evaluation of LLMs for Mental Health

Our approach to co-designing the model with mental health practitioners was instrumental in ensuring its accuracy, perceived trust, and transparency. While such approaches are emerging in the AI-HCI research [54], they remain limited. The iterative design and model evaluation through both objective measures of accuracy and qualitative practitioners’ feedback ensured that symptom detection tools are not only accurate but also clinically meaningful and ethically sensitive to the complexity of human distress. We also found merit in running 2 sets of workshops with similar procedures and a relatively small number of mental health practitioners, rather than one larger study, since significant suggestions for improvement emerged from each study. For example, the focus on risk identification and categorization prevailed in the second study, made possible only after improving the symptom categories following the first study. Thus, this iterative and staggered co-design and evaluation approach was particularly useful for embedding ethical awareness into the design and development of the DepressionSymp model.

#### Co-Designing LLMs for Symptom Identification With Cross-Validation to Strengthen Reliability

Our findings highlight that LLM-based systems may produce errors when inputs are incomplete, ambiguous, expressed defensively, or not fully disclosed, undermining assessment accuracy and practitioner trust. To strengthen reliability, we suggest contradiction-detection algorithms comparing outputs across multiple models [122,123]; once detected, contradictions should be flagged separately and trigger AI-driven prompt questions [6] requesting clarification, rephrasing, or additional detail. Prompt-based questioning [124] should similarly generate targeted follow-up questions where information is missing or only partially expressed [125], reducing ambiguity and supporting more accurate, trustworthy symptom identification. Explanation features are also needed to make reasoning transparent [35]: when a contradiction or missing information is flagged, the system should explain why, what caused the issue, and how it affects overall interpretation, with such explanations and confidence scores allowing practitioners to better evaluate output reliability and decide how much weight to place on it.

### Ethical Implications

Our findings point to 3 ethical implications.

#### Clinical Standards

The transparency categories developed in our model, including the separation of direct symptoms, indirect indicators, contextual cues, and risk, can inform the creation of clinical standards for LLM-assisted assessment [126]: this structure provides a consistent basis for clinics to adopt when reviewing written client materials before sessions, enabling more standardized case triage and clearer criteria for determining when a case requires intervention, translating our approach to symptom identification into practical guidelines that integrate AI outputs into existing assessment workflows.

#### Informed Consent

Based on our findings, consent procedures [127] should clarify that the system identifies only the client’s written language, that some symptom cues may be interpreted from indirect or implied expressions, and that all outputs are reviewed by practitioners before being used in decision-making; for example, when the system identifies social withdrawal from subtle linguistic patterns, clients need to understand that such interpretations support rather than replace clinical judgment.

#### Data Governance

Our findings highlight the need for strong data governance to ensure client privacy, security, and responsible use of sensitive information [128]: anonymizing client inputs, controlling access so only authorized practitioners can view outputs, and keeping access records can safeguard confidentiality, while secure storage and encrypted transmission protect information when saved and transmitted, and contextual information, such as cultural or linguistic details, can be recorded safely to support accurate interpretation without revealing identity.

### Limitations

The dataset has some limitations. Demographic information such as age and gender is not provided, and it may mainly represent younger individuals, as this age group is more active on social media; all posts come from Reddit, which may introduce bias in language style, such as informal expressions and vocabulary differing from clinical or other platform norms, and it is unclear whether writers are native English speakers, which may affect symptom expression and identification, making this bias hard to measure. The dataset was also annotated by the lead researcher rather than qualified mental health practitioners, potentially introducing subjective interpretation compared to expert clinical annotation, a practical decision given the challenges of recruiting clinicians for large-scale annotation of sensitive data, including limited availability and ethical governance requirements; *DSM-5* criteria and LIWC-22 were used to mitigate this by grounding the process in validated, standardized frameworks.

The study also involved a small number of mental health practitioners (6 in workshop 1 and 8 in workshop 2) due to practical constraints, limiting generalizability; participants were mainly from a younger, digitally familiar generation, further limiting generalizability to older or less digitally experienced practitioners, and the group’s differing clinical backgrounds may not capture the full diversity of clinical approaches, risking certain interpretations of symptom identification or overly increased trust in the model. However, sample sizes in qualitative co-design research vary by study aims and participant expertise, and additional participants may simply repeat similar ideas [72,129]. ROUGE-based metrics also measure lexical overlap rather than semantic correctness and cannot reliably detect negation errors, meaning a generated output that reverses the source text’s clinical meaning could still score highly, a risk partially mitigated through practitioner review of model outputs during the co-design workshops.

### Conclusions and Future Work

In conclusion, this study examined the development of DepressionSymp through an iterative co-design process with mental health practitioners, showing that practitioner involvement informed the refinement of symptom categories, supported interpretation of model outputs, and identified ethical considerations related to transparency, contextual understanding, and risk assessment, alongside challenges in cultural interpretation. These findings inform design considerations for future mental health AI systems, including support for contextual understanding of symptoms and severity, culturally sensitive interpretation of language, more nuanced risk evaluation, and human-in-the-loop oversight, as well as the importance of clinical standards, informed consent, and data governance in developing and evaluating such systems.

## Supplementary material

10.2196/95422 Multimedia Appendix 1 Participants information.

10.2196/95422 Multimedia Appendix 2 Workshop procedure and interview.

## Abbreviations

AI-HCI: AI-human-computer interaction
BLEU: bilingual evaluation understudy
CBT: cognitive behavioral therapy
DAIC-WOZ: Distress Analysis Interview Corpus – Wizard-of-Oz
*DSM-5*: *Diagnostic and Statistical Manual of Mental Disorders, Fifth Edition*
E-DAIC: Extended Distress Analysis Interview Corpus
HCI: human-computer interaction
LCS: longest common subsequence
LIWC-22: Linguistic Inquiry and Word Count
LLM: large language model
MADRS: Montgomery-Åsberg Depression Rating Scale
NLP: natural language processing
PTSD: posttraumatic stress disorder
ROUGE: recall-oriented understudy for gisting evaluation
RQ: research question
