# Supplementary material for: A Large Language Model–Based System for Depression Symptom Identification: Iterative Qualitative Co-Design Studies
Source: JMIR Form Res. 2026 Nov 3;10:e95422. doi: 10.2196/95422 (PMC13730515; doi:10.2196/95422)
Supplement: Supplementary file 1 [file formative-v10-e95422-s001.docx]

# Table 1: Participant demographics and background for Workshop 1

| **ID** | **Gender** | **Age** | **Experience (yrs)** | **Education** |
| --- | --- | --- | --- | --- |
| P1 | F | 30 | 7 | Bachelor’s Psychology and Counselling; Level 2 Counselling Skills |
| P2 | M | 35 | 10 | Bachelor’s in Psychology; Master’s in Social Work |
| P3 | F | 27 | 2 | Bachelor’s in Psychology; Master’s in Mental Health Counselling; MSc in Psychology (ongoing); Certification in Childhood Trauma |
| P4 | M | 35 | 3 | Bachelor’s in Psychology; Master’s in Counselling |
| P5 | F | 25 | 5 | Counselling Diploma; Psychotherapist qualification; Mental Health First Aid training |
| P6 | F | 28 | 5 | Bachelor’s in Psychology; MSc in Integrative Psychology (ongoing) |

# Table 2: Participant demographics and background for Workshop 2

| **ID** | **Gender** | **Age** | **Experience (yrs)** | **Education** |
| --- | --- | --- | --- | --- |
| P1 | F | 37 | 7 | Bachelor’s in Counselling and Psychotherapy |
| P2 | F | 47 | 3 | Postgraduate Diploma in Therapeutic Counselling |
| P3 | M | 28 | 6 | Bachelor’s in Psychology; Diploma in Therapeutic Counselling Skills |
| P4 | M | 41 | 5 | Master’s in Social Anthropology; Master’s in Therapeutic Counselling |
| P5 | F | 36 | 13 | Bachelor’s in Psychology and Law; Postgraduate Diploma in CBT |
| P6 | F | 32 | 8 | Bachelor of Science in Counselling and Psychotherapy; Postgraduate Certificate in Supervision in Helping Relationships |
| P7 | F | 27 | 6 | Counselling Diploma; Psychotherapist qualification; Mental Health First Aid training |
| P8 | M | 36 | 4 | Bachelor’s in Psychology; Master’s in Counselling |
