# Supplementary material for: A Large Language Model–Based System for Depression Symptom Identification: Iterative Qualitative Co-Design Studies
Source: JMIR Form Res. 2026 Nov 3;10:e95422. doi: 10.2196/95422 (PMC13730515; doi:10.2196/95422)
Supplement: Supplementary file 2 [file formative-v10-e95422-s002.docx]

DepressionSymp Workshop Procedure

**90 minutes · individual online session (Microsoft Teams)**

**Demographics (5 min)**

- Years of practice in mental health.
- Highest level of education.
- Additional mental health training.
- Age and gender identity.

# 1. Introduction (10 min)

- Explain what is DepressionSymp and how it works.
- State the aim and objective

**Manual Symptom Identification (35 min)**

- Each participant gets 5 anonymized posts (no AI output shown).
- Annotate symptoms on Miro.

# AI vs. Expert Comparison and Interview questions (40 min)

- Compare model output to the practitioner's own annotations, post by post.
- Accuracy: Do symptoms/risk align with the model? Explain/ Did the AI pick out the same symptoms and risks as you? / Where you disagreed, was it a real disagreement, or just a different way of labelling the same thing (e.g., you called it a symptom, the AI called it lifestyle)? / Does the AI's idea of how serious the case is match yours? If not, what would you look at instead?
- Trust: How comfortable would you be using this to help make a real decision about a client? / What would make you trust it more? / Is there any part of it (like the risk section) you'd trust less than the rest? Explain
- Interpretability: Can you tell why the AI put something in a certain category, without needing it explained? / Do the category names make sense to you? / Would anything help make it clearer? explain
- Ethical challenges: What are the main ethical challenges? What worries you about using a tool like this? explain
- Feedback and improvements: Do you have any suggestions for improvement? hat would you add, remove, or change? / Are there extra questions you wish the AI asked to get a fuller picture? / How could the risk section be more useful? Explain
- Is there anything we haven't asked about that feels important? / Any last concerns about how this should (or shouldn't) be used?
